# Crosstalk Between Gut Microbiota, Epigenome, and Neurotransmitters in Health and Disease

**DOI:** 10.3390/cells15141280

**Published:** 2026-07-16

**Authors:** Shabnam Nohesara, Hamid Mostafavi Abdolmaleky, Sam Thiagalingam

**Affiliations:** 1 Department of Medicine (Biomedical Genetics), Boston University Chobanian & Avedisian School of Medicine, Boston, MA 02118, USA; snohesar@bu.edu; 2 Department of Medicine, Division of Gastroenterology, Beth Israel Deaconess Medical Center, Harvard Medical School, Boston, MA 02215, USA; 3 Department of Pathology & Laboratory Medicine, Boston University Chobanian & Avedisian School of Medicine, Boston, MA 02118, USA

**Keywords:** neurotransmitters, gut microbiota, SCFAs, epigenetics, DNA methylation

## Abstract

**Highlights:**

**What are the main findings?**

- Gut microbiota has a significant influence on health by regulating the level of neurotransmitters through producing neurotransmitters directly or modulating the related metabolism pathways via modifying epigenetic mechanisms in the host.
- Gut dysbiosis is associated with disturbances in neurotransmitter production and activity, alterations in microbial metabolite profiles, and disease development through epigenetic aberrations.

**What are the implications of the main findings?**

- Profound understanding of the crosstalk between dysbiosis in the gut microbiota and the disturbance in the production and activities of neurotransmitters in the pathophysiology of diseases, especially neuropsychiatric disorders, paves the way for exploring cost-effective preventive and therapeutic interventions targeting microbial community and epigenetic aberrations.
- Gut microbiota–targeted interventions like prebiotics and probiotics may improve diseases relevant to abnormal alterations in the levels of specific neurotransmitters by mitigating epigenetic aberrations.
- Considering sex differences in GM–NTs–epigenetic interplays, it is crucial to investigate the differential therapeutic implications for sex-related diseases.

**Abstract:**

Beyond its established roles in digestion, immune regulation, and enteroendocrine signaling, the gut microbiota (GM) influences distant organs, particularly the brain, by producing or modulating the balance of neurotransmitters (NTs) and regulating their intestinal metabolism through epigenetic mechanisms, thereby shaping gut–brain communication. In this narrative review, first, we provide an overview of the major classes of NTs, such as serotonin, dopamine, GABA, glutamate, acetylcholine, epinephrine, and norepinephrine, and their functions in the body and brain. Second, we discuss the potential epigenetic mechanisms through which alterations in the levels and/or activity of NTs contribute to health and disease. Third, we explore the potential roles of the GM in the production of biologically active metabolites, such as short-chain fatty acids (SCFAs), and in the regulation of NT metabolism and function in health and disease through epigenetic mechanisms. We also highlight how GM-modulating therapies may improve disease outcomes by altering epigenetically mediated intestinal NT metabolism and availability, with downstream effects on NT activity in other organs. Finally, we discuss the current challenges and future directions for elucidating the interplay among the epigenome, the GM, and NTs.

## 1. Introduction

Neurotransmitters (NTs) are biomolecules and chemical messengers that neurons use to communicate with one another and with other organs of the body, such as muscles and glands [1,2]. They participate in numerous processes, such as regulating immune function, movement, energy balance, mood, learning, memory, sleep, and skin health [3,4,5]. The human gut microbiota (GM) is an incredibly dynamic and complex ecological system consisting of trillions of microbes specialized to the host, playing key roles in digestion, immune regulation, and overall metabolic health [6]. The GM includes bacteria, archaea, and fungi, which inhabit the mammalian gastrointestinal (GI) tract. In humans, the number of bacterial cells is approximately 3.8 × 10^13^, which roughly matches or exceeds the total number of cells in the adult body [7]. The GM also plays a key role in the production, regulating the metabolism and activity of different types of NTs, such as dopamine, serotonin, glutamate (Glu), and γ-aminobutyric acid (GABA), thereby shaping bidirectional communication between the gut and the brain, known as the gut–brain axis [8]. Although the precise pathways by which the GM modulates NT dynamics in both the GI tract and the central nervous system (CNS) remain incompletely defined, emerging evidence indicates several potential pathways and mechanisms. These include direct participation in NT biosynthesis, regulation of rate-limiting enzymes involved in NT metabolic pathways, and modulation of NT transport systems [9]. For example, the GM controls the host’s tryptophan metabolism by increasing the fraction of tryptophan available for the kynurenine pathway and diminishing the amount available for serotonin production [10]. However, the abundance of *Bifidobacterium* is linked to higher fecal GABA content in healthy human subjects [11]. Changes in NT biosynthesis and activity due to dysbiosis, an imbalance or disruption in the composition, diversity, or function of the GM, may contribute to the pathogenesis of various diseases [12]. Gut dysbiosis is a key player in the development of neuropsychiatric disorders (NPDs), such as schizophrenia (SCZ), involving several gut–brain-axis-related pathways. These include the vagus nerve, the tryptophan-kynurenine pathway, toll-like receptor (TLR) pathways, the hypothalamic–pituitary–adrenal (HPA) axis, and neurotransmitter pathways, including dopamine, serotonin, GABA, and brain-derived neurotrophic factor (BDNF), as well as short-chain fatty acids (SCFAs). For example, significant alterations in GM composition between patients with SCZ and healthy controls are associated with abnormal levels of NTs or growth factors, such as dopamine, serotonin, and BDNF [13]. In a study conducted by Clos-Garcia et al., fibromyalgia patients exhibited a remarkable elevation in the serum levels of Glu, which was linked to decreased abundances of bacteria from the *Bifidobacterium* and *Lactobacillus* genera (involved in the transformation of Glu into GABA) [14].

Ghorbani et al. reported that *Alistipes indistinctus*, *Dorea longicatena*, and *Roseburia inulinivorans* were negatively associated with dopamine levels in patients with SCZ, whereas *Roseburia intestinalis* and *Parabacteroides goldsteini* were negatively linked to serotonin and BDNF levels, respectively [13]. There is also evidence that a large part of the interactions between the GM and NTs may be mediated through epigenetic mechanisms, as certain GM-derived metabolic compounds, such as SCFAs, can function as histone deacetylase (HDAC) inhibitors, regulate microRNA (miRNA) expression, and modulate abnormalities in DNA methylation (DNAm) patterns. For instance, depressed mice exhibited noticeable reductions in acetic acid, propionic acid, pentanoic acid, norepinephrine, 5-hydroxyindoleacetic acid (5-HIAA), and serotonin (5-HT), which were associated with remarkable differences in some bacteria, particularly the genus *Allobaculum* [15].

Gut microbial dysbiosis and changes in metabolites, such as butyric acid, acetic acid, valeric acid, isobutyric acid, and isovaleric acid, affect brain NT metabolism (e.g., 5-hydroxyindoleacetic acid, threonine, kynurenine, tryptophan, and betaine aldehyde chloride) via the gut–brain axis in ASD, as well [16]. Considering these alterations, diet-based approaches or specific medications may play a potential role in determining gut health and in exacerbating or improving gastrointestinal disorders by targeting NT receptors. For example, GM–derived butyrate may offer therapeutic benefits for affective disorders characterized by altered serotonergic signaling or neuroinflammation, as it is capable of preventing oxidative stress–induced disruptions in tryptophan transport [17]. Furthermore, activation of the NT receptor dopamine receptor D2 (DRD2) in the intestinal epithelium by gut microbial metabolites exerts a protective effect against *Citrobacter rodentium*, a mouse attaching-and-effacing (AE) food-borne pathogen responsible for infections such as gastroenteritis and enterocolitis [18]. In another study, administration of a high-sugar and high-fat (HSHF) diet induced gut dysbiosis, disrupted the GI tract, and disturbed neurotransmitter metabolism in both the intestine and the brain, ultimately leading to alterations in brain function and circRNA expression profiles [19]. The GM–derived metabolite trimethylamine N-oxide (TMAO) was also demonstrated to degrade certain circRNAs, and baseline GM composition determined the conversion rate of choline to TMAO. Moreover, altered levels of *Candida albicans* and/or *Klebsiella pneumoniae* could affect neurotransmitter secretion and the cholinergic system [19]. Although substantial progress has been made in elucidating the role of the microbiota–gut–brain axis in NT modulation, as well as in the development of gut microbiota-targeted therapeutics for diseases associated with dysregulated NT levels and gut dysbiosis, a focused literature review devoted specifically to recent advances in this field, with particular emphasis on the potential role of epigenetic mechanisms, is still lacking. Accordingly, a comprehensive review of the current state of the art in GM-targeted therapeutics is warranted.

This review integrates current evidence on the role of the GM in modulating NT metabolism and function in health and disease through epigenetic pathways. Here, we (i) summarize the major NT classes and their physiological roles, (ii) describe epigenetic mechanisms by which changes in NT levels and activity influence disease processes in diverse psychiatric disorders, (iii) evaluate how GM-derived factors regulate NT metabolism and signaling via epigenetic regulation, and (iv) discuss the therapeutic potential of microbiota-derived metabolites and microbiota-targeted interventions to restore intestinal NT balance and influence NT activity in the brain and peripheral organs. Finally, we outline key challenges and future research directions in unraveling the complex interactions among the epigenome, the GM, and NTs.

## 2. Methods

In this narrative review, we summarized current evidence on the interactions among NTs, the GM, and epigenetic mechanisms, with a particular focus on their implications for human health and disease pathogenesis. The objective was to integrate findings from diverse experimental and clinical studies rather than to perform a formal systematic review or meta-analysis.

A comprehensive literature search was conducted using four electronic databases: PubMed, Web of Science, Scopus, and Embase. The search included articles published between January 2003 and May 2026. Search terms combined keywords related to different NTs and the GM with major epigenetic mechanisms using Boolean operators. Representative search terms included “neurotransmitter” OR “gut microbiota” OR “gut microbiome” combined with “DNA methylation,” “histone modification,” “histone acetylation,” “microRNA,” “miRNA,” and “epigenetic”. Additional searches incorporated terms related to GM-based interventions, including probiotics, prebiotics, synbiotics, postbiotics, fecal microbiota transplantation (FMT), and dietary interventions. Reference lists of relevant publications were also screened to identify additional studies.

The retrieved literature was screened for relevance based on titles and abstracts, followed by full-text assessment of potentially eligible publications. Original experimental studies, observational studies, and clinical investigations examining the relationships among NTs, GM, and epigenetic regulation were prioritized. In addition, authoritative review articles were consulted to provide background information, summarize established concepts, and identify seminal primary studies. Editorials, conference abstracts lacking sufficient methodological detail, and publications unrelated to the scope of this review were excluded.

Rather than applying predefined systematic review eligibility criteria or formal quality assessment tools, the selected literature was evaluated for scientific relevance, methodological rigor, and contribution to the understanding of GM–NT–epigenetic interactions. More than 150 relevant publications were included in the final synthesis. The findings were organized narratively according to major NT systems, epigenetic mechanisms, GM-derived metabolites, GM-based interventions, and their roles in physiological regulation and disease pathogenesis, with emphasis on identifying common mechanistic pathways, emerging evidence, and current knowledge gaps.

## 3. Overview of Key Neurotransmitters Involved in Psycho-Pathogenesis

### 3.1. Serotonin

5-Hydroxytryptamine (5-HT, serotonin), a multifunctional bioactive agent, is derived from tryptophan and capable of acting as an NT within both the central and peripheral nervous systems, and as a peripheral hormone secreted by platelets [20,21]. Interestingly, the GI tract is responsible for the production of over 90% of the body’s serotonin [22]. Serotonin secreted by enterochromaffin cells (ECs) in the gut participates in various GI functions, including vasodilation, peristalsis, and the perception of pain and nausea through stimulation of serotonin receptors on intrinsic and extrinsic sensory nerve fibers located within the lamina propria [23]. Serotonin also plays a powerful role in neural signaling, and disturbances in its production or reuptake are associated with the development and progression of psychiatric disorders [24]. In addition to regulating neuronal function in the CNS, serotonin has the ability to influence pain perception, platelet aggregation, mucosal homeostasis, electrolyte transport, and smooth muscle function in peripheral tissues [25]. Serotonin contributes to the motility and contractility of the gut, airways, uterus, and vasculature, either directly or via enteric and autonomic neural pathways as well [26]. The first step in its synthesis in the body involves hydroxylation of dietary tryptophan, followed by decarboxylation to produce active serotonin. The serotonin transporter (SLC6A4, SERT) plays a key role in preserving optimal levels of serotonin by actively importing and facilitating its degradation by intracellular monoamine oxidases [27].

Serotonin is a key player in the pathogenesis of numerous neurological, psychiatric, and systemic diseases, such as migraine, carcinoid syndrome, and GI disorders, via a wide array of receptor subtypes [28]. It is also capable of acting as a paracrine signaling molecule and a growth-promoting agent. Serotonergic receptors in both the cerebral tissue and the GI tract are considered key pharmacological targets for designing new compounds to regulate serotonin-dependent signaling [29].

### 3.2. Dopamine

Dopamine is a NT derived from the amino acid tyrosine, which itself can be synthesized from dietary phenylalanine. Although dopaminergic neurons comprise less than 1% of the brain’s cellular population, dopamine accounts for approximately 80% of the catecholamines present in the CNS. It is a master regulator in both the peripheral and central nervous systems that acts via five types of G-protein-coupled receptors (D1–D5 dopamine receptors). Dopaminergic neurotransmission affects a wide range of functions, including cognition, motivation, satisfaction, reward processing, eating behaviors, motor control, emotion, sleep, voluntary movement, the regulation of glucose balance, and the maintenance of renal, retinal, and cardiovascular homeostasis [30,31]. Maintaining dopamine at optimal levels contributes to healthy brain function and helps prevent the development of NPDs. Dopamine receptors are present on almost all immune cells, where dopamine affects various processes, such as T-cell activation, antigen presentation, and inflammation [32,33]. In the brain, dopamine is mainly produced by neuronal cells, whereas the majority of dopamine in the body is produced in peripheral tissues. The primary biosynthetic pathway for dopamine in the human brain is the “l-phenylalanine (Phe) → l-tyrosine (Tyr) → (s)-3,4-dihydroxyphenylalanine (levodopa, l-dopa) → dopamine (Phe–Tyr–dopa–dopamine)” metabolic pathway.

### 3.3. Glutamate (Glu)

Glu is one of the non-essential amino acids and the primary excitatory NT in the neural network, contributing to nearly 60% of all neurotransmission activity. Despite the presence of large amounts of Glu in the brain, the extracellular fluid contains only a small fraction of this molecule, and the highest levels are found inside nerve terminals. Proper regulation of Glu in the intracellular space prevents excessive stimulation of postsynaptic Glu receptors and facilitates efficient signaling that protects neurons against excitotoxic damage. Astrocytes play an important role in maintaining Glu homeostasis in the CNS by regulating the balance between Glu uptake and release [34]. L-glutamic acid (L-Glu) plays a key role in several physiological processes, such as neuronal communication and CNS plasticity, which regulate normal brain function. Glu is capable of activating N-methyl-D-aspartate (NMDA) receptors, which modulate synaptic functions relevant to learning, memory, and cognition. Glu has also been shown to be involved in the regulation of cellular energy metabolism via its conversion to α-ketoglutarate, a key intermediate in the tricarboxylic acid cycle, and contributes to ammonia detoxification through its transformation into glutamine in astrocytes. Glu is also the precursor of γ-aminobutyric acid (GABA), the principal inhibitory neurotransmitter involved in regulating excitatory signaling and maintaining brain stability.

### 3.4. GABA

GABA is a non-proteinogenic amino acid and the principal inhibitory NT present in the mammalian CNS, and it is synthesized through the irreversible decarboxylation of glutamate [35]. In addition to being found in the nervous system of vertebrates, GABA is naturally found in some plants, grains, fruits, vegetables, and fermented products [36,37]. Human GABA levels decrease with age (approximately 5% per decade, with a faster decline in women), which may accelerate the development of cognitive deficits and brain aging [38,39]. Therefore, GABA-containing nutritional supplements may contribute to the alleviation of cognitive deficits, as well as depression [40], hypertension [41], diabetes [42], and may also modulate immune function [43,44].

### 3.5. Acetylcholine (ACh)

Acetylcholine (ACh) is a classical NT that acts as a chemical messenger in neuronal cells to communicate with other cells. ACh is a key neurotransmitter in the nervous system, facilitating communication between nerve cells and muscles. It contributes to the initiation of muscle contraction at neuromuscular junctions [45], regulates diverse immune cell functions (T cells, B cells, and macrophages) [46], promotes learning and memory in the brain [47], and modulates some involuntary body functions like heart rate [48], digestion [49], and the release of glandular products via the autonomic nervous system [50]. ACh is formed from choline and acetyl-CoA via a reaction mediated by choline acetyltransferase (ChAT).

### 3.6. Norepinephrine and Epinephrine

Norepinephrine and epinephrine are catecholamine NTs derived from the amino acid tyrosine via dopamine as an intermediate (Tyrosine → L-DOPA → dopamine → norepinephrine → epinephrine) and act as key players in the body’s stress response. Both of these NTs influence adrenergic receptors and subsequently affect cardiovascular function, arousal, and alertness. Norepinephrine is primarily generated and released by neuronal cells located in the locus coeruleus of the brain. The locus coeruleus contains almost 20,000 norepinephrine-producing neurons that send widespread projections throughout several brain areas. Norepinephrine participates in diverse physiological processes, including attention regulation, fear responses, working memory, spatial learning, fear-associated memory, auditory fear conditioning, memory retrieval, and spatial reference processing [51]. However, epinephrine is predominantly synthesized as both an NT and a hormone in the adrenal medulla and is mainly involved in the human body’s “fight-or-flight” response by elevating heart rate, releasing stored energy, promoting energy mobilization, and improving vasodilation in skeletal muscles to ensure appropriate responses to sudden stress [52].

## 4. Epigenetic Regulation of NTs and Their Receptors in Health and Disease

There are several epigenetic mechanisms that may regulate NT signaling. DNA methylation, histone modifications, and miRNA-mediated regulation are among the best-known epigenetic mechanisms involved in these processes.

### 4.1. DNA Methylation (DNAm)

DNAm is an epigenetic modification in which a methyl group (–CH_3_) is incorporated into DNA, usually at the cytosine base at CpG or CpA sites, without altering the DNA sequence [53]. DNAm (which mostly contributes to gene silencing) is one of the epigenetic mechanisms involved in regulating the dynamics of NT production and release in the synaptic cleft [54]. For example, dynamic changes in DNAm can regulate genes associated with glutamate receptor signaling and Glu uptake in Bergmann glial cells [55]. Interestingly, short-term AMPA receptor activation itself can induce hypomethylation of specific regions of the chGLAST promoter, indicating active demethylation mechanisms that stimulate sustained transporter expression during periods of high excitatory activity [55]. In another study, hypomethylation of genes relevant to glial Glu transporters elevated the glial Glu transporter protein levels and uptake activity in primary cultures of Bergmann glia cells from the chick cerebellum and in Müller glia cell line derived from the human retina [56]. Synaptic upscaling, an adjustment that enhances excitatory signaling, is also linked to reduced DNAm, which elevates the expression of genes encoding Glu receptors and the proteins involved in trafficking these receptors to the synaptic membrane in cortical pyramidal neurons harvested from rats [57]. In human studies, highly stressed students exhibited stress-related epigenetic alterations in dopamine and serotonin transporter genes. Bellia et al. found that highly stressed students had greater DAT1 methylation, particularly those with the 9/9 genotype (9/9 repeats of a variable number tandem repeat), and greater miR-491 levels, which decrease DAT1 expression. Conversely, one specific CpG methylation site in the serotonin transporter was more prevalent in low-stress individuals, and miR-135 was lower in high-stress students. Collectively, the authors concluded that these patterns of stress-related epigenetic alterations in dopamine- and serotonin-related genes may serve as potential biomarkers of stress susceptibility in sensitive young adults [58]. DNAm patterns in the serotonin transporter gene (SLC6A4) and the tryptophan hydroxylase 2 gene (TPH2) are also considered potential predictive biomarkers to determine how patients may respond to treatment during disease [59]. For example, Mohammadi et al. reported that treatment of subjects with major depressive disorder (MDD) with serotonin reuptake inhibitors could improve clinical symptoms by decreasing DNAm at the promoter CpG sites of several genes implicated in depression, including BDNF, NR3C1, FKBP5, and SLC6A4 [60]. Table 1 summarizes additional studies on the interactions between DNAm and NTs across various diseases.

### 4.2. Post-Translational Modifications of Histone Proteins

Histone modifications are epigenetic alterations involving the covalent addition or removal of chemical groups from histone proteins around which DNA is wrapped. These modifications influence chromatin structure and modulate gene expression without changing the underlying DNA sequence [72,73,74]. Protein monoaminylation is another biochemical modification in which biogenic monoamines, including serotonin, dopamine, and histamine, are capable of covalent binding to specific protein targets [75,76,77]. This reaction is mediated by transglutaminase 2, an enzyme that contributes to the transamidation of primary amines to the γ-carboxamide groups of glutamine residues. Protein monoaminylation is linked to a broad spectrum of physiological functions, including blood clot formation, platelet activation, and the regulation of G-protein–mediated signaling pathways. Histone proteins, most notably histone H3 at glutamine residue 5 (H3Q5), have recently been identified as additional monoaminylation targets in vivo. Monoaminylation at H3Q5 has been shown to be a key regulator of chromatin structure and the transcriptional activity of genes [78]. For example, a study by Farrelly et al. indicated that serotonylation of H3Q5 contributes to the recruitment of TFIID, acting cooperatively with neighboring H3K4 trimethylation in the mouse brain and cultured human serotonergic cells [79]. The HDAC inhibitors butyrate and trichostatin A (TSA) have been shown to reduce serotonin transporter (SERT) expression by boosting the binding between acetylated histone H3 or H4 and the hSERTp1 region upstream of exon 1a, leading to diminished promoter activity in Caco-2 cells [80]. Notably, selective inhibition of HDAC2, but not HDAC1, recapitulates the effects of butyrate and TSA in lowering SERT expression [80]. A recent study showed that serotonin regulates histone acetylation by modulating the balance between histone acetyltransferases and deacetylases and hence affects gene expression and embryonic development in early porcine embryos [81]. In this context, serotonin is capable of upregulating KAT8 and downregulating HDAC2 and HDAC3 at the 4-cell stage, which in turn results in increased histone acetylation. On the contrary, at the blastocyst stage, serotonin elevates HDAC1 and HDAC3 expression while decreasing KAT8 activity, leading to reduced histone acetylation [81]. Chan et al. reported that serotonin transporter-dependent histone serotonylation in the placenta of mice facilitates the neurodevelopmental transcriptome [82]. Table 2 summarizes studies on diverse features of histone modification–NT interactions in different diseases.

### 4.3. MicroRNAs

miRNAs are a class of small non-coding RNA molecules (approximately 20–23 nucleotides) that are capable of regulating gene expression [92,93,94]. Recently, it has been uncovered that almost 70% of identified miRNAs are expressed in the nervous system, where their expression patterns usually exhibit strong spatial and temporal specificity [95]. miRNAs have demonstrated the ability to regulate genes encoding NTs or their receptors. For example, the miRNA-15a, miRNA-15b and miRNA-16 suppress the dopamine D1 receptor (DRD1) expression by targeting the 3′UTR from −12 to +154 bp in HEK293, U87, SK-N-SH and SH-SY5Y cell lines [96]. Another study showed that miR-181a acts as a potential modulator of mammalian AMPA-type Glu receptors, with possible roles in mediating drug-induced changes in synaptic plasticity in adult mice [97]. Widmark et al. also found that edited miR-376b-3p expression promoted an increase in intracellular GABA and augmented cell surface availability of GABA type A receptors in the human neuroblastoma cell line SHSY5Y [98]. Bhupender et al. found that elevated expression of miR-502-3p decreased the GABA current and general GABA function in mouse hippocampal neurons, indicating a negative relationship between miR-502-3p levels and GABAergic synapse function [99]. Furthermore, Yang et al. reported that Glu/GABA homeostasis regulated by miR-8-5p can control the behavioral changes between solitary and gregarious locusts [100]. Table 3 provides a summary of studies examining interactions between miRNAs and NTs across various diseases.

## 5. Neurotransmitters and Gut Microbiota

Substantial alterations in the composition of gut microbes influence the synthesis of NTs, since the GI tract and gut microbes are responsible for their production and activity alongside brain tissue [110]. This section will discuss how the digestive system, gut microbes, and their metabolites influence the biosynthesis, activity, and availability of NTs.

### 5.1. Serotonin and Gut Microbiota

Because serotonin is synthesized in both the gut and the brain, derangements in the abundance of gut microbes and gut function may disrupt its production, potentially increasing the risk of NPDs and diverse cognitive deficits. The GM can modulate host serotonin systems through both direct and indirect mechanisms. Studies using germ-free or antibiotic-treated animals have identified direct mechanisms involving altered expression of key genes related to serotonin biosynthesis. Indirectly, gut microbes also produce a wide range of metabolites capable of regulating host serotonergic signaling via multiple pathways [111]. On the other hand, serotonin transporter deficiency has been found to be associated with the initiation of gut dysbiosis and alterations in the metabolic function of the GM in mice. In this line, serotonin transporter (SERT) −/− mice exhibited substantial alterations in the GM, including an increased abundance of *Bacilli* species (including *Lactobacillus*, *Streptococcus*, *Enterococcus*, and *Listeria*) and a reduced abundance of commensal microbes (*Bifidobacterium* and *Akkermansia muciniphila*), which were connected to changes in the expression of host lipid metabolism–related genes [112].

### 5.2. Dopamine and Gut Microbiota

Certain gut bacteria have the capacity to synthesize dopamine or its precursors, thereby contributing to peripheral dopamine availability. 3-Methoxytyramine (3MT) has been found to be a key precursor for dopamine biosynthesis by gut bacteria. In the host, 3MT is synthesized through the O-methylation of dopamine by catechol-O-methyltransferase (COMT), a reaction that reduces endogenous dopamine availability. Gut microbes are capable of producing dopamine by O-demethylating 3MT. Given that 3MT is produced when host COMT diminishes dopamine levels, gut bacteria capable of converting 3MT into dopamine could counteract COMT-mediated dopamine catabolism [113]. This process may be associated with epigenetic shifts since 3MT elevates acetate production by *E. limosum* and *B. producta*, and acetate is a metabolite with known epigenetic activity [113].

### 5.3. Glutamate and Gut Microbiota

The gut bacteria play a key role in Glu biosynthesis and modulating its availability via both microbial and host-mediated pathways [114]. Fermentation of Glu by some anaerobic bacteria, such as *Clostridium kluyveri* and related species, results in the production of epigenetic metabolites like acetate and butyrate as core end products, along with ammonia and CO_2_; however, their epigenetic contribution remains hypothetical and has not yet been demonstrated. Glu is considered a key nitrogen source for certain gut bacteria, and various species use, synthesize, or secrete Glu in diverse ways. Therefore, alterations in GM composition are associated with changes in Glu levels in the intestine and the blood. For example, *Bacteroides thetaiotaomicron* is capable of elevating the amount of Glu, since it possesses certain enzymes that contribute to the synthesis of Glu from basic nitrogen sources and metabolites [115]. It also contributes to the biosynthesis and secretion of free Glu into the gut by breaking down dietary proteins and participating in normal nitrogen metabolism. Alterations in the GM composition during certain diseases may influence glutamate levels in plasma and feces. For example, obese subjects exhibited an increased abundance of *Streptococaceae RA*, which was negatively associated with the fecal glutamate/glutamine ratio [116].

### 5.4. γ-Aminobutyric Acid (GABA) and Gut Microbiota

GABA is also produced by gut microbes in the colon and affects neural signaling within the enteric nervous system, which in turn contributes to brain function and behavior [117,118]. GABA is capable of modulating enteric neuron activity and regulating intestinal functions by influencing GABAA and GABAB receptors [119]. Activation of GABAergic signaling, especially via the GABA *B* receptor, mitigates LPS-induced enteritis by suppressing oxidative stress and inflammation through inhibition of the TLR4/MyD88/NLRP3 pathway, improving antioxidant defenses, and restoring the balance of the GM characterized by increased abundance of *Lactobacillus*, a butyrate-producing bacterium, and a decreased abundance of pathogenic bacteria, predominantly *Enterobacteriaceae* [120].

GABA, also referred to as a natural “neuro-vitamin”, is a key regulator of the GM and, hence, brain function and host behavior in health and disease, potentially via epigenetic mechanisms. For example, high levels of GABA supplementation in an in vitro fecal slurry fermentation model substantially elevated the relative abundance of the *Actinobacteria phylum* (particularly through increased levels of *Bifidobacterium*) and enhanced the production of acetic acid through microbial fermentation [121].

### 5.5. Ach and Gut Microbiota

Gut bacteria can indirectly influence ACh levels by modulating choline availability, the key precursor for acetylcholine synthesis. Certain microbial communities influence choline metabolism and subsequently alter ACh synthesis in the enteric environment and the CNS. Stress-related gut dysmotility is associated with lowered colonic ACh levels, whereas pharmacological activation of nicotinic ACh receptors (nAChR) hampers these effects [122]. ACh or ACh-producing bacteria, such as *L. plantarum,* are capable of elevating gut contractions and promoting gut motility in *Drosophila* larvae [123]. Reduced levels of choline and its metabolites, including ACh and phosphatidylcholine (PC), have been found within the colon of individuals with IBD [124].

In brain diseases such as AD, Amyloid-β (Aβ) can interfere with the vagus nerve–mediated cholinergic anti-inflammatory pathway by inhibiting the expression of M1 muscarinic ACh receptors (M1 mAChRs) in the brain. Disruption of central cholinergic tone may also impair vagus nerve–mediated anti-inflammatory signaling, which leads to reduced expression of choline acetyltransferase (ChAT) and decreased ACh synthesis in the gut of AD mice [125]. The reduction in ACh availability consequently alters the activation of the α7 nicotinic ACh receptor (α7 nAChR) expressed on intestinal macrophages, which in turn contributes to the shift in macrophages toward a pro-inflammatory state by stimulating NF-κB signaling. This inflammatory shift gives rise to enteric neuronal damage, causes the intestinal epithelial barrier dysfunction, and elevates the activation of amyloidogenic pathways within the gut [125]. Notably, daily ethanol injection for three consecutive days in mice is also associated with rapid upregulation of α7 nAChR in the limbic system and the elevated expression of TNF-α, IL-1β, and ITGβ2 across multiple brain regions. Interestingly, gut dysbiosis emerges only after a few days of such alcohol exposure and is characterized by an increased abundance of the *Prevotellaceae* family, suggesting that early brain inflammatory and cholinergic alterations may contribute to the subsequent development of gut dysbiosis [126].

### 5.6. Norepinephrine, Epinephrine, and Gut Microbiota

Individuals with irritable bowel syndrome (IBS) exhibit decreased plasma NT levels, including serotonin and norepinephrine, and plasma norepinephrine levels are negatively correlated with gut bacterial alpha diversity in these patients [127]. More specifically, norepinephrine is positively associated with the *Bacteroidetes* phylum and negatively linked to the *Firmicutes* phylum [127]. The disturbance of neurotransmitter metabolism in chronic restraint stress (CRS)-induced depressive conditions is also linked to GM dysbiosis and inflammation. CRS mice exhibit diminished concentrations of plasma serotonin, dopamine, and norepinephrine associated with GM dysbiosis, characterized by a considerable elevation in the abundance of *Helicobacter*, *Lactobacillus*, and *Oscillibacter* and a reduction in the abundance of *Parabacteroides*, *Ruminococcus*, and Prevotella abundance [128]. *Helicobacter*, *Lactobacillus*, and *Oscillibacter* are positively linked to depressive behaviors but are negatively associated with neurotransmitter metabolism. In contrast, *Parabacteroides* and *Ruminococcus* are negatively associated with depressive behaviors but are positively connected to neurotransmitter metabolism [128]. Norepinephrine is also capable of direct interaction with QseC, thereby activating the quorum-sensing signaling pathway in *F. nucleatum* bacteria and elevating the expression of virulence-associated genes (*FadA*, *FomA*, and *Fap2*), which in turn give rise to increased bacterial invasiveness in vitro. At the same time, norepinephrine contributes to the translocation of *F. nucleatum* into the intestinal tissue, reducing the abundance of SCFA–producing bacteria (*Prevotellaceae* and *Lactobacillaceae*), increasing the concentrations of host pro-inflammatory mediators (IL-6 and IL-1β), and eventually exacerbating colonic inflammation in a mouse model of IBD [129].

## 6. Microbiota-Based Interventions

GM-based interventions can modulate NT systems through epigenetic mechanisms. These interventions, including probiotics, prebiotics, specific diets, and FMT, alter GM composition and metabolite production. Microbial metabolites, especially SCFAs, and tryptophan derivatives may also affect epigenetic processes such as DNAm, histone modifications, and non-coding RNA regulation. Through these mechanisms, GM-based interventions regulate the expression of genes involved in the secretion of NTs and receptor signaling, thereby influencing serotonergic, dopaminergic, glutamatergic, and cholinergic pathways.

### 6.1. Postbiotics

Postbiotics are bioactive metabolites (e.g., SCFAs, indole derivatives, and microbial peptides and polysaccharides) released by gut microbes that play key roles in numerous biological events [130,131]. These include restoring the opioidergic and endocannabinergic pathway and enhancing the expression of genes involved in gut barrier integrity, antioxidant and anti-inflammatory actions, and immune tolerance through epigenetic mechanisms involving the elevation of histone acetylation, relaxing chromatin structure, and regulation of miRNA expression [132,133,134,135]. Resident gut bacteria, via their metabolites, especially SCFAs, can also stimulate ECs to increase serotonin synthesis, hence affecting gut motility and serotonergic homeostasis [136]. SCFAs have also demonstrated the ability to activate TPH1 transcription in a human model of ECs, indicating a mechanistic link between these metabolites and serotonin synthesis [136]. In a human intestinal cell line (Caco-2/TC7), Buey et al. reported that SCFAs, including acetate, propionate, and butyrate (individually and in combination), are capable of modulating the serotonin transporter function and expression and regulating the expression of key serotonin receptors (5-HT1A, 5-HT2B, and 5-HT7) [137]. However, elevated levels of butyrate after short-term consumption of a high-fat diet (HFD) in mice reduce intestinal serotonin availability by increasing its uptake by ileal cells, which may contribute to improving gastrointestinal adverse effects following radiation therapy [138]. In a mouse model, Wang et al. also reported that, while allergic inflammation (Th2/Th17-driven) decreases serotonin production in both ECs and enteric serotonergic neurons, serotonin deficiency is associated with alterations in the GM and altered lipid metabolism, particularly a marked decrease in the microbial metabolite isovaleric acid. They further showed that the administration of isovaleric acid restored serotonin synthesis and gut motility in mice [139]. Although isovaleric acid is known as an epigenetic modifier, its epigenetic effects remain hypothetical and have not been demonstrated in this study.

The microbial metabolite acetate also stimulates TPH1 expression, coding a key enzyme involved in serotonin production, in both IPEC-J2 and RIN-14B cells in pigs, possibly through the activation of FFAR3 receptors [140]. Even though acetate is a well-established epigenetic regulator, its potential epigenetic contribution to this effect lacks direct experimental validation. Nevertheless, acetate-producing bacteria, especially *Lactobacillus amylovorus* and its metabolites, are capable of enhancing serotonin biosynthesis, signifying a specific microbe–host interplay that regulates serotonin production [140]. Furthermore, gut commensal microbes and their metabolites, particularly SCFAs, have been found to be key players in the sympathoadrenal epinephrine response to acute hypoglycemia. Among the other beneficial outcomes of oral SCFA supplementation is its involvement in stress-related epinephrine release, potentially mediated by certain mechanisms such as enhanced catecholamine biosynthesis. Supplementation with metabolites like acetate and butyrate during antibiotic treatment can also improve both tonic and stress-induced epinephrine release in mice [141]. However, whether epigenetic mechanisms underlie this effect remains to be elucidated experimentally.

In another interesting study, Wolugbom Jr et al. found that polycystic ovarian syndrome (PCOS)-associated depression in female virgin Wistar rats, accompanied by neuroinflammation, increased ACh levels, and elevated expression of HDAC2 in the PFC and hippocampus, was reversed by 21 days of oral gavage with acetate [142]. GM-derived metabolites like butyrate and indole-3-propionic acid could also influence Glu release by human astrocytes, which plays key roles in health and disease, possibly via epigenetic mechanisms [143]. This proposition is supported by another study demonstrating that valproate and sodium butyrate attenuated manganese (Mn)-induced locomotor deficits in mice by increasing astrocytic Glu transporter 1 (GLT-1) expression through histone deacetylase inhibition [144].

Regarding the other beneficial effects of SCFAs, another study demonstrated that nine months of dietary SCFA supplementation (a mixture of sodium acetate, butyrate, and propionate) in APP/PS1 mice not only reshaped the GM but also diminished Aβ deposition and tau hyperphosphorylation and improved cognition. These effects were mediated by modulation of the Glu-glutamine shuttle acting on glutamine synthetase and enhancing astrocyte-neuron metabolic coupling to reduce oxidative damage [145]. Xu et al. also found that sodium butyrate (NaB) could alleviate motor dysfunction and attenuate dopaminergic neuron loss in MPTP-induced PD mice by increasing brain NTs, such as serotonin and dopamine levels in the striatum, as well as reducing systemic inflammation via suppression of NF-κB signaling, improving GPR109A expression and the intestinal barrier function through the elevation of tight junction proteins and the reduction in gut permeability [146]. Similarly, Guo et al. reported that the beneficial effects of NaB against dopaminergic neuron loss in MPTP-induced PD mice are associated with elevated striatal NT levels, mitigating the overactivation of glial cells, reducing striatal pro-inflammatory mediators, inhibiting the TLR4/MyD88/NF-kB pathway in the colon and striatum, and improving intestinal barrier dysfunction [147]. Furthermore, using an MPTP-induced PD mouse model, Duan et al. found that butyrate supplementation could improve sleep disturbances by restoring the levels of dopamine and its metabolite 3,4-dihydroxyphenylacetic acid (DOPAC) in the striatum through targeting the BDNF-TrkB pathway [148]. However, experimental evidence supporting an epigenetic mechanism underlying these effects remains to be established.

Table 4 summarizes additional studies investigating the effects of GM-derived metabolites on NT secretion or synthesis. It is important to note that, although substantial evidence supports the role of postbiotics and microbial metabolites in modulating NTs, their effects are highly context-dependent and vary with the specific metabolite, its concentration, exposure duration, tissue examined, disease state, and experimental model. Therefore, some of the apparent discrepancies among studies are likely attributable to differences in biological context rather than to true inconsistencies.

**Figure 1:**
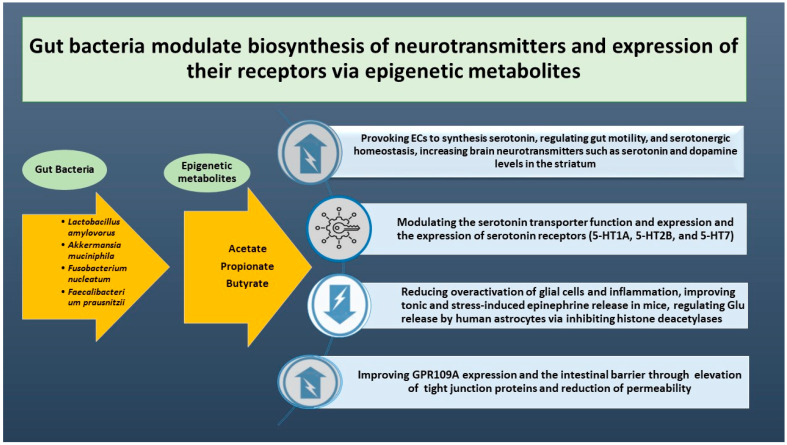
Effects of metabolites produced by gut bacteria on the regulation of the biosynthesis of neurotransmitters and the expression of their receptors, likely via epigenetic mechanisms. SCFA-producing bacteria of the human gut microbiota, such as *Lactobacillus amylovorus*, *Akkermansia muciniphila*, *Fusobacterium nucleatum*, and *Faecalibacterium prausnitzii* contribute to generating metabolites like acetate, propionate, and butyrate. These metabolites, in turn, modulate the biosynthesis of neurotransmitters and the expression of their receptors, possibly through epigenetic mechanisms.

Figure 1 shows how the production of epigenetic metabolites by gut bacteria may regulate the biosynthesis of neurotransmitters and the expression of their receptors.

### 6.2. Probiotics

Probiotics are live microorganisms capable of exerting health benefits on the host when they are administered in adequate amounts [158,159]. Resident gut bacteria such as *Akkermansia muciniphila* and *Faecalibacterium prausnitzii*, as well as their extracellular vehicles (EVs) involved in the production of epigenetic metabolites, have demonstrated the ability to regulate serotonin-dependent pathways in human intestinal epithelial (Caco-2) cells [160]. In this study, EVs derived from both bacterial species could enhance serotonin concentrations and modulate core genes involved in serotonin synthesis and regulation [160]. In another study, a combination therapy with probiotics and fructo-oligosaccharide (FOS) in children with ASD could alleviate disease severity and gastrointestinal symptoms. These effects were mediated by an increased abundance of commensal bacteria (*Bifidobacteriales* and *B. longum*), preventing the overgrowth of suspected pathogenic bacteria (*Clostridium*), and increasing the concentration of epigenetic metabolites like acetic acid, propionic acid, and butyric acid, while reversing the hyperserotonergic state (elevated serotonin) and dopamine metabolism dysfunction (reduced homovanillic acid) [161]. Using a MPTP-induced PD mouse model, Srivastav et al. also found that a probiotic cocktail containing *Lactobacillus rhamnosus GG*, *Bifidobacterium animalis lactis*, and *Lactobacillus acidophilus* could hamper MPTP-induced neuronal loss in the nigrostriatal pathway by reducing the expression of monoamine oxidase (MAO) B in the striatum, and elevating the content of neurotrophic factors and butyrate in the brain [162]. Based on another report, *Fusobacterium nucleatum* in the guts of flies could improve the psychomotor effects of amphetamine by elevating brain dopamine transporter levels [163]. Their findings indicated that the butyrate metabolite produced by *Fusobacterium nucleatum* is capable of increasing the expression of the gene encoding the dopamine transporter through HDAC1 inhibition, thereby improving dopamine-motivated behaviors.

Probiotics can be employed to protect against dopamine neurotoxicity by mitigating epigenetic aberrations, such as modifying histone acetylation through alterations in metabolite levels involved in the modulation of neuroinflammation and neuronal survival. As an interesting example, Xu et al. found that exogenous supplementation of *A. muciniphila* alleviated 6-OHDA-induced motor impairment and dopamine neuronal damage, while reducing microglial activation and neuroinflammation by increasing butyrate concentration in both the intestine and brain of rats [164].

Gut microbes have also demonstrated the ability to improve NPDs by producing GABA, upregulating GABA receptor gene expression, and influencing the oxidant–antioxidant balance [165,166,167,168]. For example, Sabna BS et al. found that *Enterococcus faecium* BS5 represents a viable strain for industrial GABA production and functional fermented food formulation [169]. In addition, *Bifidobacterium dentium* is capable of encoding enzymes for GABA biosynthesis from Glu, glutamine, and succinate. This was shown both in vitro, through GABA release in defined media, and in vivo, where mice exhibited elevated fecal GABA levels compared with germ-free controls [170]. Notably, while GABA-producing *Lactobacillus* and *Bifidobacterium* strains have shown promising efficacy in alleviating depressive-like behaviors in BALB/c mice [171], norepinephrine can also influence probiotic efficiency and bacterial behavior, primarily through its effects on microbial abundance and the gut microenvironment. For example, Niu et al. found that norepinephrine promoted the growth of most *Levilactobacillus* strains but decreased their antimicrobial activity, indicating its potential role in reducing the probiotic efficacy of *Levilactobacillus* strains [172]. Although these studies indirectly suggest the involvement of epigenetic mechanisms in the effects of probiotics, additional experimental evidence is needed to establish their direct epigenetic actions.

### 6.3. Fecal Microbiota Transplantation

FMT is a medical approach in which processed stool from a healthy donor is transferred into the GI tract of a recipient to treat disease (or, in an experimental setting, to induce disease) by reshaping the GM composition and modulating NT biosynthesis, potentially through epigenetic mechanisms [173,174,175]. For example, it has been shown that ASD-FMT mice exhibited low SERT and 5-HT1AR expression levels and high TPH1 expression levels in the colon and increased expression levels of TPH2 and SERT in the prefrontal cortex versus typically developing-FMT mice [176]. Li et al. found that 4 weeks of FMT treatment reduced serotonin and GABA concentrations in the serum, while increasing dopamine levels in the serum in children with ASD [177].

Sun et al. found that FMT could improve GI transit in rats with high-fat diet-induced obesity by reducing small intestinal serotonin concentration, TPH1 expression, deoxycholic acid, and cholic acid [178]. Wang et al. observed that FMT improved VPA-induced ASD in mice by modulating the serotonergic and glutamatergic synapse signaling pathways, which were associated with the successful engraftment of SCFA-producing bacteria, including *Turicibacter* and *Alistipes* [179]. Li et al. reported that FMT improved alcohol-induced depression-like behaviors in C57BL/6J mice by stabilizing intestinal barrier integrity (increasing the expression of the intestinal tight junction proteins ZO-1 and occludin) and elevating serotonin content in both intestinal and brain tissues [180].

### 6.4. Prebiotics

Prebiotics are dietary components (typically non-digestible fibers or substrates) that are utilized by gut microbes and beneficially modulate the GM by targeting cellular signaling pathways and regulating NTs [181,182]. The GM depends on dietary components, host-derived substrates, and naturally occurring compounds to influence its community composition, metabolic functions, and interactions with host physiology. Certain prebiotics have been shown to promote the growth of NT-producing bacteria [183]. For example, consumption of short-chain fructooligosaccharides (scFOS) or inulin for two weeks increased the relative abundances of GABA-synthesizing bacteria (based on 16S rRNA sequencing), and luminal GABA concentrations in the ileum and colon of mice [184]. In a study conducted by Braga et al., prebiotic FOS and Aspergillus-derived enzymes could increase gut and brain GABA concentrations by influencing certain species like *Parabateroides*, *Akkermansia*, *Muribaculum*, *Hungatella*, *Marvinbryantia*, *Flavonifractor*, and *Incertae_sedis* in adolescent mice [185]. Xie et al. found that a high-cellulose diet could improve intestinal motility by enhancing intestinal homeostasis and promoting enteric nervous system performance through elevating acetate production and the subsequent suppression of HDAC3 in the mouse colon [186]. They concluded that a cellulose-rich diet or acetate supplementation may be considered a promising option to improve disruptions in intestinal motility and brain function.

Herbal medicine prescriptions like Tongxie yaofang and Shugan decoction, as well as flavonoid and polyphenol compounds, may be considered potential prebiotic-like compounds for improving diseases by regulating the serotonin pathway through increasing the specific bacterial abundance (such as *Firmicutes* and *Bacteroidetes*) involved in producing epigenetic metabolites [187].

Beneficial effects of prebiotics may be associated with the regulation of hippocampal serotonin levels, potentially mediated by modulating miRNA expression as well. For example, morphine-induced gut dysbiosis is linked to changes in the expression of fecal miR-129-5p. In a study conducted by Li et al., the authors found that morphine could increase fecal miR-129-5p in mice, which in turn inhibits *Bacteroides vulgatus* (*B. vulgatus*) growth by targeting the bacterial gene BVU_RS11835 and elevating hippocampal serotonin levels [188]. Their results also demonstrated that ginsenoside Rg1, a type of steroid glycoside and triterpene saponin present in ginseng, attenuated morphine addiction by inhibiting miR-129-5p, enhancing the level of *B. vulgatus*, and decreasing serotonin levels. Dong et al. reported that neferine, a natural compound and a bisbenzylisoquinoline alkaloid derived from the seed embryo of lotus, mitigated the depressive-like behaviors by elevating the levels of antidepressant NTs (dopamine, serotonin, and norepinephrine) and attenuating the hippocampal damage in depressed mice via restoring *Lactobacillus* levels in the intestinal microbiota [189]. Prebiotic inulin has also been shown to alleviate anxiety and depression-like behaviors in mice undergoing alcohol withdrawal by elevating the abundance of butyrate-producing bacteria, including *Faecalibacterium* and *Roseburia*, increasing SCFA production, and modulating serotonin metabolism [190]. Additionally, using “a defined human commensal consortium grown in anaerobic bioreactors”, Horvath et al. found that saffron reshaped amino acid metabolism by reducing tryptophan and increasing tryptamine and indole-3-acetic acid. It also increased GABA, glutamate, glycine, and dopamine but decreased L-DOPA, tyrosine, and anthranilic acid, in addition to shifting SCFA profiles, including increasing formic and isobutyric acids and reducing propionic and valeric acids [191]. Cheng et al. found that berberine, a benzylisoquinoline alkaloid present in various plants in the *Berberis* genus, including barberry, Oregon grape, and goldenseal, improved CRS-induced depression-like behaviors by enhancing the levels of BDNF and serotonin, suppressing inflammation, and restoring gut microbial diversity and SCFA concentrations (fold-change: acetate 1.8-fold, butyrate 2.2-fold) [192].

**Figure 2:**
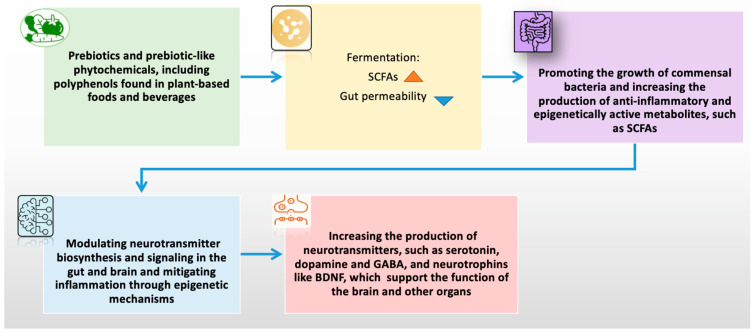
Effects of prebiotics and prebiotic-like compounds on the regulation of neurotransmitter biosynthesis and signaling in the gut and brain. Prebiotics and prebiotic-like compounds present in plant-based foods modulate neurotransmitter biosynthesis and signaling in the gut and brain and reduce inflammation by enhancing the growth of beneficial bacteria and increasing the production of anti-inflammatory and epigenetically active metabolites, such as short-chain fatty acids (SCFAs).

Yang et al. reported that an isoflavone-enriched diet could attenuate motor deficits and dopaminergic neuron loss in MPTP-induced PD mice by enhancing the expansion of intestinal *Lactobacillus*, particularly *Lactobacillus intestinalis*, and increasing serotonin levels in the serum and brain. In their study, serotonin elevation increased activation of 5-HT1A receptor-mediated PI3K–AKT signaling and inhibited ferroptosis, a form of programmed cell death [193]. Figure 2 shows how prebiotics and prebiotic-like compounds found in plant-based foods contribute to modulating NT biosynthesis and signaling in the gut and brain and mitigating inflammation by enhancing the growth of beneficial bacteria and increasing the production of anti-inflammatory and epigenetically active metabolites, such as SCFAs.

**Figure 3:**
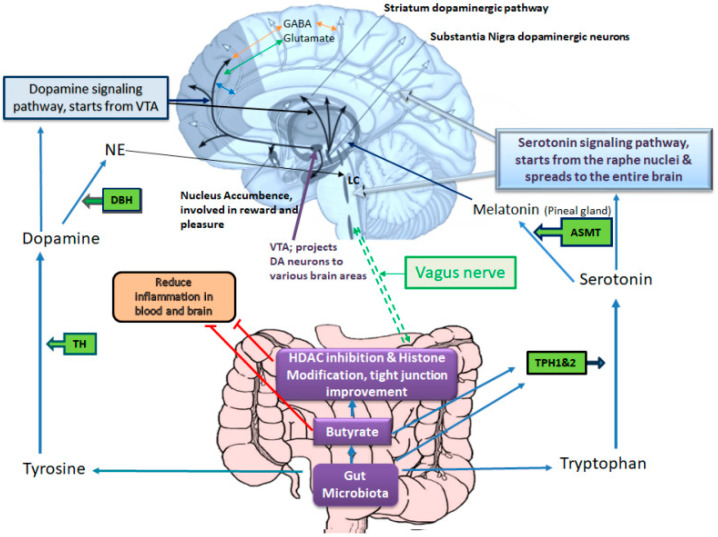
Gut microbiota–host interplay in NT metabolism involved in neuropsychiatric diseases. Disruptions in brain serotonin, dopamine, norepinephrine, glutamate and GABA may induce neuropsychiatric diseases. The serotonin signaling pathway starts from the raphe nucleus and spreads throughout the brain, positively influencing the nucleus accumbens, which is responsible for reward and pleasure. The enzymes TPH1 and TPH2 are involved in serotonin synthesis, and TH is involved in dopamine synthesis. Serotonin and dopamine can further be converted into melatonin and noradrenaline, respectively. In brain tissue, these neurotransmitters interact with glutamate (excitatory) and GABA (inhibitory) neurotransmitters, which are also implicated in the pathogenesis of neuropsychiatric disorders. The gut microbiota and its metabolites play a key role in providing substrates and influencing enzymes involved in dopamine and serotonin synthesis, consequently affecting the production of noradrenaline (particularly in the locus coeruleus, LC) and melatonin (in the pineal gland) production. Melatonin regulates circadian rhythms, which are often disrupted in major neuropsychiatric disorders. Additionally, the gut microbiota and its metabolites, especially butyrate, help mitigate blood and brain inflammation and influence the activity of the vagus nerve, which directly communicates with the brain through the medulla oblongata. The red T-shape symbols indicate inhibition. This figure is adapted from our previous study published by MDPI [200].

A ketogenic diet (KD) is another remedy to modulate NTs and gut microbiota. Ketone bodies were shown to possess broad neuromodulatory actions and are capable of changing NT levels in both healthy and diseased brains [194,195]. The increased β-hydroxybutyric acid (BHB) produced by the KD might be linked to alterations in brain GABA and Glu levels in male rats, possibly through altering the expression levels of the GABA-T and GAD67 genes [196]. In a study conducted by Orlando et al., the KD could also promote the gut–brain axis in a rat model of IBS by downregulating the elevated mucosal serotonin without influencing its transporter and receptor levels, while promoting brain BDNF levels, leading to a compensatory reduction in TrkB (a BDNF receptor) to maintain a physiological steady state [197]. Moreover, Liu et al. found that KD treatment could enhance the concentration of serotonin, adenylate cyclase, cyclic adenosine monophosphate (cAMP), and norepinephrine in brain tissue and the expression of DRD1, DAT, PKA, DARPP32, and cAMP at the protein level in male spontaneously hypertensive rats (SHR) and Wistar Kyoto (WKY) rats. These alterations were mediated through elevating the richness and diversity of the GM, increasing the abundance of *Ruminococcus_gauvreauii*_group, *Bacteroides, Bifidobacterium*, and *Blautia,* and reducing the abundance of *Lactobacillus*, *Romboutsia*, *Facklamia*, and *Turicibacter* [198]. Qiao et al. reported that mechanistically, BHB produced during a KD inhibits HDAC1 and HDAC2, and subsequently increases H3K27 acetylation and the transcriptional upregulation of SIRT4 and Glu decarboxylase 1 (GAD1) [199]. Their data indicated that BHB-induced SIRT4 de-carbamylated and inactivated Glu dehydrogenase, thereby conserving Glu for GABA synthesis. This increased GAD1 expression elevated the GABA-to-Glu ratio in the brain, ultimately reducing neuronal excitability and seizures in a PTZ-induced acute seizure mouse model [199]. Figure 3 summarizes the concepts discussed regarding the relationship between the GM, its epigenetic metabolites, and NT metabolism linked to neuropsychiatric diseases.

## 7. Sex Differences in GM–NTs–Epigenetic Interactions

Sex is known as a biological variable capable of influencing different aspects of human health and disease [201]. Accumulating evidence indicates that sex may be considered one of the most important biological factors capable of affecting GM composition, NT metabolism, and epigenetic modifications along the gut–brain axis [110]. Sex differences in stress-induced behavior may be associated with alterations in monoaminergic neurotransmission, hyperactivity of the HPA axis, inflammatory processes, GM composition, and brain–gut metabolic interactions [202]. Therefore, therapies based on modifying GM composition for disease treatment may differ between sexes in the future, owing to sex-specific differences in GM profiles and fecal metabolite patterns observed in animal models of disease [203]. Sex hormones can influence the gut–brain axis at multiple levels, including the central nervous system, the enteric nervous system, and enteroendocrine cells [204]. Sex differences have also been found to be involved in NT metabolism and neuroimmune responses such as glial cell activation and cytokine production, likely explaining sex-based differences in triggering neuroinflammation and behavioral abnormalities [205]. Furthermore, sex hormones may directly regulate serotonergic, dopaminergic, GABAergic, and glutamatergic signaling pathways and shape microbial diversity and metabolite production. For example, testosterone administration is capable of preserving certain SCFA-producing taxa involved in producing metabolites and supporting bioenergetic pathways in response to severe energy deficit in healthy, physically active men [206]. Estrogens also contribute to modulating the release of neurotransmitters like serotonin, noradrenaline, and dopamine, which are considered important mediators of their antidepressant effects [207]. Sex hormones can also influence inflammatory actions in both the central and enteric nervous systems by reducing bacterial diversity, exerting beneficial effects on the intestinal microbiota, and regulating motility and exocrine function. On the other hand, gut microbes may affect the metabolism and recirculation of sex hormones through microbial enzymes such as β-glucuronidases. In particular, the GM plays a key role in modulating systemic estrogen levels via a mechanism named the estrobolome, and hence gut dysbiosis gives rise to dysfunction in this process. In this line, one study reported a dysfunctional estrobolome in APP/PS1 transgenic female mice marked by a reduced abundance of *Limosilactobacillus* and *Lactobacillus*, an elevated abundance of *Ligilactobacillus*, lower butyrate production, decreased activity of the β-glucuronidase enzyme in fecal samples, low bioavailability of estradiol, a disrupted estrous cycle, and cognitive impairments associated with gut dysbiosis [208]. Nevertheless, gut microbes and their metabolites may improve behavioral abnormalities associated with sex [209,210]. Metabolites produced by the GM, such as butyric acid, may modulate appetite and hormone levels in the hypothalamus of PCOS rats through the gut–brain–ovary axis, contributing to regulating the expression of ovarian steroidogenic factors and enhancing follicular development, granulosa cell function, and reproductive performance [211]. Butyrate can also contribute to improving obesity-induced dysfunctions in spermatogenesis in male mice fed a high-fat diet [212]. These bidirectional interplays contribute to sex-specific immune responses, neuroinflammation, and susceptibility to neuropsychiatric and metabolic diseases. Epigenetic mechanisms, including DNAm and histone modifications, further mediate sexually dimorphic modulation of microglial activation and NT-dependent gene expression. Collectively, understanding sex differences in GM–NT–epigenetic interplay is crucial, particularly when considering the potential therapeutic applications for disorders such as anxiety and depression.

## 8. Conclusions and Future Perspectives

Accumulating evidence indicates that the GM is a master regulator of NT biology and contributes to bidirectional communication between the gut and distant organs, particularly the brain. Gut microorganisms can influence the synthesis, availability, and signaling of NTs such as serotonin, GABA, dopamine, and related precursors through multiple mechanisms, including the production of bioactive metabolites, modulation of immune pathways, regulation of intestinal permeability, and activation of neural routes such as the vagus nerve. Owing to the production of a large proportion of the body’s serotonin in the gut, GM-dependent pathways may influence metabolism, mental health, sleep, gastrointestinal motility, digestion, and immune homeostasis. Emerging evidence further suggests that certain microbial metabolites may affect epigenetic processes that regulate NT-related genes and receptors in the brain and other body organs, with potential implications for therapeutic interventions.

Despite these promising findings, several important limitations and caveats must be acknowledged. First, much of the current evidence remains associative rather than causal, and many studies are based on animal models whose GM composition, immune function, and neurobiology differ considerably from those of humans. Second, in probiotic studies, findings are often heterogeneous and modest in magnitude, possibly reflecting differences in bacterial strains, dosing regimens, the period of administration, baseline GM composition, diet, medication use, age, sex, and other host-specific factors. Third, while fecal FMT has shown promising outcomes in experimental studies, its clinical translation is difficult due to donor–recipient mismatch, variable microbial engraftment, the uncertain identification of key therapeutic organisms or metabolites, and safety concerns owing to the potential transfer of pathogens or undesirable traits. Fourth, evidence supporting prebiotics, postbiotics, and dietary interventions is still largely based on preclinical studies, and there are no robust human data. Moreover, measuring NTs in peripheral samples does not necessarily reflect neurotransmission within specific brain regions, making the interpretation of mechanistic studies challenging. These limitations exhibit the need for carefully designed mechanistic studies and well-powered, double-blind randomized controlled trials that can clarify whether GM-induced changes in NT synthesis, availability, receptor signaling, or epigenetic regulation directly contribute to clinical improvement in neurological, psychiatric, metabolic, and gastrointestinal disorders. Future studies should focus on implementing larger and more translational animal models, standardized protocols for GM sampling and analysis, longitudinal designs, and appropriately matched healthy control groups.

A feasible translational pathway for GM-based interventions should be based on rigorous preclinical models aimed at the detection of specific microbial strains, microbial communities, metabolites, or diet-sensitive pathways that meaningfully affect NT biology. Patient-specific iPSC-derived brain organoids may also provide a promising platform for investigating the effects of gut-derived metabolites on epigenetic modulation of neurotransmitter systems. These efforts should integrate germ-free and humanized models with multi-omics techniques such as metabolomics, transcriptomics, epigenomics, and immune profiling, followed by mechanistic validation to clarify actionable targets and predictive biomarkers. Finally, precision-microbiome strategies tailored to individual host characteristics may prove more effective than one-size-fits-all interventions.

Collectively, the gut–brain axis is now well recognized as a core biological communication network, but the specific NT and epigenetic mechanisms that mediate GM-dependent health and disease remain incompletely defined. The field is advancing rapidly, yet substantial work is still required to move from intriguing associations to reproducible, mechanism-based, and clinically effective GM-targeted therapies.

## Figures and Tables

**Table 1:** NTs’ aberrant DNAm across various diseases.

Gene Encoding a Neurotransmitter or Its Receptor	Disease/DNA Methylation Detection Technique	Population/Sample	Key Outcomes vs. Controls	Ref.
Dopamine D2 and D1 receptor (Drd2 and Drd1)	Social defeat stress (SDS)/pyrosequencing	Mice/brain	DNA hypomethylation of Drd2 in the prefrontal cortex and amygdala of mice with SDS	[61]
Dopamine transporter gene (DAT1)	Addiction to nicotine/methylation-specific PCR assay	137 nicotine addicts, 274 addicts, 105 “sports subjects”, and 290 controls/peripheral blood	Increased methylated CpG islands in addicted subjects (~41%), nicotine-dependent (~63%), and athletic group (~66%) vs. controls (~42%)	[62]
DRD2 gene	Psychosis/pyrosequencing	82 psychotic patients and 57 age and sex-matched controls/saliva	DNA hyper-methylation of DRD2 in psychotics	[63]
Serotonin transporter (SLC6A4) and serotonin receptor (HTR2A)	Maternal gestational diabetes (GDM)/pyrosequencing	90 pregnant women/placenta	DNA hyper-methylation of placental SLC6A4 in the GDM group	[64]
SLC6A4	Dystonia/quantitative bisulfite pyrosequencing	Subjects with cervical (*n* = 49), myoclonus (*n* = 41) and dopa-responsive dystonia (*n* = 27) and controls (*n* = 56)/blood samples	A relationship between the percentage of methylation at several sites of the SLCA64 promoter in subjects with (dopa-responsive) dystonia	[65]
5-HTTLPR	Panic disorder (Korean descent)/pyrosequencing	232 with panic disorder and 93 controls/superior longitudinal fasciculus	Lower levels of DNA methylation at 5 CpG sites of 5-HTTLPR	[66]
Vesicular Glutamate Transporters (Vglut)	Early life stress (ELS) and/or ethanol consumption/Targeted next-generation bisulfite sequencing	Rats/ventral tegmental area (VTA), nucleus accumbens (NAc), dorsal striatum, and medial prefrontal cortex	Vglut1-2 CpG-specific hypomethylation in VTA and lower promoter and higher intronic Vglut3 methylation in the NAc of ethanol-drinking rats exposed to ELS	[67]
γ-aminobutyric acid (GABA)A receptor α1 subunit	Suicide/major depressive disorder (MDD)/bisulfite mapping	Human/postmortem brains of those who died by suicide and controls with almost equal age	Promoter DNA hypermethylation of GABAA receptor α1 subunit gene (3 CpG sites) in patients who died by suicide	[68]
GABAAR	Alcohol use disorder (AUD)/methyl-DNA-immunoprecipitation (MeDIP)	20 control and 20 AUD subjects/postmortem brain tissue	Promoter DNA hyper-methylation in the cerebellum and a decrease in the α2 subunit expression	[69]
GABBR1	AUD/targeted bisulfite Sanger sequencing	17 individuals with AUD pathology (13 male) and 31 controls (21 male)/blood and brain	Sex-specific effects of AUD on GABBR1 promoter methylation; hypomethylation in AUD in the amygdala and the mammillary bodies in men	[70]
Norepinephrine transporter (NET)	Attention deficit hyperactivity disorder (ADHD)/Sequenom MassARRAY EpiTYPER analysis	23 adults with ADHD and 23 controls	NET promoter hyper-methylation (“region 1”) in ADHD vs. controls; methylation of several sites in this region is inversely linked to the severity of hyperactivity–impulsivity symptoms	[71]

**Table 2:** Histone modification–NT interactions in different diseases.

Neurotransmitter or Its Receptor	Type of Histone Modification/Disease/Histone Modification Detection Technique	Population/Sample	Key Outcomes	Ref.
Serotonin and 5-HT3	Histone acetylation/alcohol use disorders (AUDs)/fluorometric assay	Human SK–N–MC and neurons treated with ethanol	Elevated 5-HT3 and HDAC-1 & 3 expression, and an increase in HDAC activity after ethanol treatment	[83]
5-HT1A receptor (HTR1A)	Histone acetylation/depression/Western blotting	Mice/hippocampus	Histone acetylation coupled with HTR1A is involved in depression pathogenesis	[84]
Serotonin	Histone serotonylation/major depressive disorder (MDD)/Western blotting, Chromatin Immunoprecipitation (ChIP), ChIP-sequencing (ChIP-seq), and Immunofluorescence	Mice exposed to chronic social defeat stress and MDD subjects/dorsal raphe nucleus (DRN)	Key role of H3K4me3Q5ser in stress-mediated transcriptional plasticity; dysregulated H3K4me3Q5ser dynamics in DRN	[85]
Dopamine	Histone H3 dopaminylation/substance use disorder/Western blotting	Male rats/ventral tegmental area (VTA)	H3Q5dop Accumulation in rats’ VTA subject to abstinence from heroin self-administration	[86]
Dopamine	Histone H3 dopaminylation/substance use disorder/Western blotting	Male rats/nucleus accumbens (NAc) and medial prefrontal cortex (mPFC)	Acute and prolonged accumulation of H3Q5dop in NAc contributes to cocaine-seeking behavior during cocaine abstinence	[87]
Glutamate transporter-1 (GLT-1)	Histone acetylation/peripheral nerve injury/Western blotting	Rats/spinal astrocytes	Spinal nerve ligation increases HDAC2 and reduces GLT-1 in spinal astrocytes	[88]
GABAA receptor (GABA_A_R)	Histone acetylation/alcohol dependence/chromatin immunoprecipitation followed by quantitative PCR (ChIP–qPCR)	Rats’ cortex and mPFC	Chronic ethanol exposure and withdrawal upregulate HDAC2 and HDAC3 linked to the Gabra1 promoter, reducing its H3 acetylation and GABAAR α1 subunit expression	[89]
Norepinephrine	Histone acetylation/Parkinson’s disease/ChIP	6-OHDA-induced cell damage in primary cultures from rat ventral mesencephalon and a dopaminergic cell line (MN9D)	Norepinephrine upregulates tyrosine hydroxylase (TH) and BDNF protein levels by increasing TH promoter H4 acetylation	[90]
Norepinephrine transporter (NET)	Histone acetylation/hypertension and depression/Western blotting and Chip-PCR	Depression-hypertensive patients/leukocytes from blood	Lower levels of histone acetyltransferase (HAT) and H3K27ac in patients vs. controls; higher acetylation elevates NER expression	[91]

**Table 3:** The interactions between miRNAs and NTs in various diseases.

Gene Encoding a Neurotransmitter or Its Receptor	Type of miRNA/Disease/MiRNA Detection Method	Population/Sample	Key Outcomes	Ref.
Serotonin reuptake transporter (SERT)	miR-24/irritable bowel syndrome (IBS)/quantitative real-time RT-PCR (qRT-PCR)	IBS patients and mice/human intestinal mucosa epithelial cells of the colon	Upregulation of miR-24 and downregulation of SERT in intestinal mucosa epithelial cells in IBS subjects vs. controls	[101]
Serotonin pathway genes, including Slc6a4 and Htr1a	miR-320-5p/early-life stress–induced depressive behavior/qRT-PCR	Rats/frontal cortex	Reduction in miR-320-5p is linked to increased Htr1a expression	[102]
Tryptophan hydroxylase 2 (TPH2)	miR-669g/aggression and memory deficits/qRT-PCR	Mice/brain	Increased brain miR-669g expression reduces TPH2 expression, and cerebral serotonin levels, increasing aggression and memory impairment	[103]
Dopamine transporter gene (DAT) and tyrosine hydroxylase (TH)	miR-133b/Parkinson’s disease (PD)/qRT-PCR	Human and mouse/brain	Association between reduced expression of miR-133b in midbrain of subjects with PD and reduced expression of DAT and TH	[104]
TH	miRNA-325-3p, miR-326-3p, and miR-330-5p/mania/qRT-PCR and luciferase reporter assay to assess direct binding activity of miRNAs to the 3′-UTR of *Th* gene	72 h REM sleep-deprived (SD) rats/prefrontal cortex	TH up-regulation and reduced expression of miRNA-325-3p, miR-326-3p, and miR-330-5p	[105]
Glutamate decarboxylase-67, vesicular GABA transporter, and GABA transporter-3	miRNA-15b-5p, miRNA-144-3p, miRNA-582-5p, and miRNA-879-5p/chronic unpredictable mild stress (CUMS)-induced depression/qRT-PCR	Mice/cortex	Up-regulation of miRNA-15b-5p, miRNA-144-3p, miRNA-582-5p and miRNA-879-5p reduced the expression of glutamate decarboxylase-67, vesicular GABA transporter and GABA transporter-3	[106]
GABAA receptor (α5GABAAR), gephyrin, and dystrophin	miR-30a, miR-31, miR-190a, and miR-190b/anesthetics-induced recognition and working memory dysfunction/qRT-PCR	Aged rats/mPFC and hippocampus	Reduced levels of miR-30a, miR-31, miR-190a, and miR-190b are linked to increased expression of α5GABAAR, gephyrin, and dystrophin	[107]
Glutamate receptor subunits GluR2 and NR2B	miR-223/ischemic stroke/qRT-PCR	Mice/brain	MiR-223 deficiency increases NR2B and GluR2, NMDA-induced calcium influx, and miniature excitatory post-synaptic currents in the hippocampal neurons, and hence cell death	[108]
Norepinephrine, serotonin, and dopamine	miR-323a-3p/CUMS-induced depression/qRT-PCR	Mice/hippocampal tissue	Knockdown of miR-323a-3p reduced depression-like behaviors and restored monoamine neurotransmitter levels	[109]

**Table 4:** Effects of GM metabolites on NT secretion.

Microbial Metabolite	Neurotransmitter Affected/Direction of Effect	Experimental Model or Disease Context/Proposed Mechanism	Key Caveats/Explanation for Contradictory Findings	Ref.
Propionate	Norepinephrine, dopamine, and GABA/↑ or ↓ based on dose	CUMS rats/low-dose administration exerted antidepressant effects by restoring NT levels	A dose-dependent biphasic (hormetic) effect/Low-dose (2 mg/kg/day) produces antidepressant effects, whereas high-dose (200 mg/kg/day) exacerbates depressive-like behaviors.	[149]
Acetate and propionate	Dopamine and norepinephrine/↑	Acute carbon monoxide poisoning in rats/improving the reduced amounts of GPR41, GPR43, dopamine, and norepinephrine in the hippocampus	SCFA effects are disease-, dose-, and composition-dependent	[150]
Propionate	Dopamine/↑	Mitochondrial toxin-induced neurotoxicity in MN9D dopaminergic cells/suppressing oxidative stress and maintaining the expression of enzymes involved in the catecholamine biosynthesis pathway	In vitro limitation, dose dependence, indirect mechanism, and model specificity	[151]
Indole-3-acetic acid (IAA)	Serotonin/↑	Sleep deprivation in mice/improving cognitive impairments and restoring intestinal integrity by binding to the aryl hydrocarbon receptor (AhR) in enterochromaffin cells (and hence enhancing the secretion of serotonin	Indirect mechanism; IAA increased gut-derived serotonin via EC–AhR signaling in an SD-specific model	[152]
Butyrate	Serotonin/↑	Human P-STS enteroendocrine cell line/influencing acetylcholine-induced Ca^2+^ signaling and serotonin secretion	Acute in vitro effect; receptor-independent and concentration-dependent, limiting in vivo extrapolation	[153]
Deoxycholate (secondary bile acid)	Serotonin/↓ (indirect)	Human P-STS enteroendocrine cell line/acetylcholine-induced Ca^2+^ signaling and serotonin secretion	Acute in vitro effect; indirect inhibition via muscarinic signaling, not TGR5-mediated	[153]
Butyrate	Dopamine/↑	1-methyl-4-phenyl-1,2,3,6-tetrahydropyridine (MPTP)-induced PD mouse model/restoring normal sleep architecture in PD mice by increasing dopamine through the BDNF-TrkB pathway	Model-specific; butyrate restored dopamine but not acetylcholine, indicating selective neurotransmitter modulation	[148]
Butyrate	Serotonin/↑	A microfluidic tri-culture model capable of enabling directional signaling among colonic epithelial cells, sensory neurons, and microbial metabolites/Butyrate elevated epithelial serotonin secretion by influencing epithelial GPR41 or GPR43	Context-dependent effects influenced by dose, disease state, and experimental model	[154]
Butyrate	GABA/↑	Chronic Unpredictable Mild Stress in rats/sodium butyrate improved the behavioral abnormalities and inflammatory responses by restoring hippocampal BDNF/TrkB/CREB signaling	Butyrate-associated effects may depend on disease context, microbiota composition, and host metabolism	[155]
Butyrate	GABA/↑	The Gnal+/− mouse model of DYT25 dystonia/butyrate reduced motor deficits by regulating neurotransmitter pathways, particularly GABA signaling in the striatum	Model-specific; butyrate restored GABA, while dopamine and acetylcholine were unchanged	[156]
Butyrate	Dopamine, glutamic acid, GABA, glutamine/↑	d-galactose-induced aging mice model/improving cognitive behavior and brain histopathology, decreasing GFAP, IBA-1, Aβ, AChE, MDA, IL-6, IL-1β, and TNF-α levels, and enhancing BDNF, PSD-5, GSH-Px, and SOD levels	Multifactorial intervention; NT changes cannot be attributed solely to butyrate	[157]

## Data Availability

No new data were created or analyzed in this study. Data sharing is not applicable to this article.
